# The puzzle of intersectoral collaboration and health. Revisiting implementation research

**DOI:** 10.1093/heapol/czae075

**Published:** 2024-11-18

**Authors:** Daniel Maceira, Stephanie M Topp

**Affiliations:** Economics Department (UBA), University of Buenos Aires, Cordoba 2122, Buenos Aires 1113, Argentina; Researcher National Council of Scientific Research (CONICET) and Center for the Study of State and Society (CEDES), Sanchez de Bustamante 27, Buenos Aires 1173, Argentina; Board Member and Chair for the Americas, Health Systems Global (HSG); Director MBA Health, University of San Andrés, 1276, Buenos Aires 1116, Argentina; College of Public Health Medical and Veterinary Sciences, James Cook University, James Cook Dr, Townsville, Queensland 4811, Australia; Nossal Institute, University of Melbourne, Unit 2/32 Lincoln Square N, Melbourne, Carlton VIC 3053, Australia; Health Systems Global (HSG)

**Keywords:** Intersectoral collaboration, health systems global, governance

Advancing the field of health systems research requires drawing from diverse bodies of knowledge. This involves not only deepening expertise within our own intellectual domains but also engaging with the research interests, experiential knowledge and skills of others. It requires a commitment to understanding different perspectives, and seeking opportunities for cross-fertilization and knowledge expansion.

This is not a simple task. We have been trained, as social scientists, to go in-depth into our area of specialization, narrow down our research questions, identify and separate effects to better understand how the world works, how policies affect health results and how behaviours change over time when exposed to different stimulus. We are often focused on whether and how institutions become resilient to external change, and how community efforts affect and are affected by the policymaking process. The age-old debates about the merits and limitations of qualitative and quantitative approaches, and how best to combine them, often form a backdrop to this work.

Nevertheless, there is a common understanding—we believe—that interdisciplinary efforts ‘are’ valuable, with cross-disciplinary spillovers enriching research and strengthening our capacity to understand, engage with and ultimately change reality. Health Systems Global, a society committed to advancing research on health policy and systems was founded on this very principle: bringing together the parts of the whole for learning, exploration and even transformation. Yet the complexity of this endeavour increases when extending beyond the boundaries of the health care system, to include the social, cultural and political determinants of health and consider how coordination across all social sectors can be achieved.

COVID-19 highlighted the importance of coordinating efforts to foster collaboration in designing, implementing and evaluating public policies and of building a consistent agenda to address large-scale (national to global) health risks. The pandemic shone a light on the concept of preparedness in the face of public health emergencies, demonstrating that effective responses were multi-level and cross-sectoral in nature—requiring high-quality social interventions, public–private partnerships, community participation and dialogues spanning income, labour, transportation, education and health initiatives.

Furthermore, the COVID crisis demonstrated the need to reimagine the role of external assistance in the context of broader intersectoral development goals and to better understand how regional efforts can strengthen collective responses to health emergencies, including the production of goods and services and information technologies. The pandemic also accentuated the need to revisit the role of international organizations and societies, including Health Systems Global, as testing grounds for partnerships between research, government, non-government and private sector entities and forums for robust debates that transcend traditional geographic or sectoral boundaries.

This special issue of Health Policy and Planning aims to contribute to this growing but still nascent area of knowledge on intersectoral collaboration in health. Supported by the International Development Research Center of Canada, this series is part of the legacy of Health Systems Global Symposium organized in Bogotá 2022 (HSG, 2022). Intersectoral collaboration in health was one of the four subthemes of the event, focusing on exploring experiences and methodological approaches to how health systems (should) interact with other social policies, such as nutrition, environment, water and sanitation and education. The discussions also examined how different levels of government—national, provincial and local—coordinate in the provision of care and how to address the segmentation of health care systems that challenges intersectoral collaboration, and the governance of the health sector as a whole. The Symposium showcased work focused on (then-ongoing) pandemic policies with valuable learnings for the future.

Following a call for papers, eight articles were selected through a peer-review process. This volume consolidates a rich variety of views, each complementing the others, showing how much we have learnt but also how much more research is required in this subfield.

Half of the eight selected articles selected focus on case studies at the national or subnational levels, each employing different methodologies and offering unique perspectives on intersectoral analysis. Beare *et al*. (2024) examines the intersection between health and social environment in Uganda, providing an interesting programme-control process evaluation of two service delivery modalities—one involving an NGO facility and the other a hospital consortium. The study employs a range of methods—including interviews with community members and health staff, focus groups and data analysis. Similarly, Brahmachari *et al*. (2024) investigates the interaction between community ties and the health care sector, focusing on the performance of informal primary health care services in rural India. Using a Social Network Analysis approach, the article delves into the practices and relationships of 34 informal providers, with the intention of understanding the relevance of local social networks in enhancing quality of care.

The insights from Beare *et al*. and Brahmachari *et al*. converge on several key learnings. First, they demonstrate the effectiveness of using process evaluation to measure social ties and prompt integration between providers and patients, contributing to a virtuous circle to enhance quality and coverage. Second, they highlight the potential for achieving institutional sustainability through informal and community-based ties. Finally, they underscore the importance of trust and engagement mechanisms as crucial factors for creating impactful services.

Next, Yasobant *et al*. (2024) explores intersectoral collaboration in India during the pandemic, focusing on how different areas of the government at Ahmedabad interacted with each other, and with private stakeholders to improve response to COVID. The article draws on more than 50 interviews with a broad range of actors, identifying cooperation mechanisms that enhanced preparedness to the crisis. It also shows the challenges posed by a fragmented organizational structure and offers insights for a comparative analysis of resilience and priority-setting criteria during an emergency.

Also at the local level, but with an emphasis on a specific policy implementation process, Silumbwe *et al*. (2024) present an integrative framework for understanding the dynamics of tobacco control in Zambia. As with the first two articles, this qualitative study, which involved 27 informants, highlights the importance of sectoral representation and authority in implementing effective public health interventions, even, and perhaps more importantly, in circumstances where there is an absence of local evidence. The study concludes that clear policies, trust and political will are essential ingredients for successful policy implementation, particularly when behavioural factors beyond the reach of health system structures influence a program’s effectiveness.

Two comparative studies were selected for this special issue. The first, by Baum *et al*. (2024) seeks to identify effective drivers of COVID-19 response through an institutional analysis of intersectoral actions. The study applies the ideas–interests–institutions framework across 16 countries with varying levels of economic development, exploring commonalities in their emergency response strategies. Despite differences at the national level, the authors emphasize common features that align with other papers in this series, including the need for empathetic and socially engaged political leadership, transparent decisions and equitable responses as part of building intersectoral support and trust.

While Baum *et al*. (2024) examine commonalities in intersectoral responses to COVID-19, Joshi *et al*. (2024) shift the focus to non-communicable diseases in low- and middle-income countries, exploring how active policy engagement can be facilitated in the context of research initiatives, before being scaled up. The authors highlight several factors important for building sustainable partnerships for cross-sectoral policies and programmes, including the importance of policy co-creators who understand the policy landscape, are engaged with the implementation process, and are willing to champion their programs. Similar to several of the other papers in this issue, Joshi *et al*. stress that successful cross-sector collaboration depends on three critical conditions—strong leadership, a cooperative environment and trust in the strategic direction of the policy.

Following the empirical studies, the special issue closes with two conceptual papers, grounded in extensive literature reviews, which offer analytical frameworks for understanding intersectoral collaboration in health, and broaden the discussion to include environmental interactions and health workforce responsiveness. The first paper by Broghi *et al*. (2024) focuses on the interaction between health and environment, with the authors finding still persistent gaps in the knowledge base around cross-sectoral coordination, not only in designing intersectoral policies but also in implementing strategic co-financing efforts, developing appropriate skills for these kinds of initiatives, and improving monitoring and costing mechanisms. Meanwhile, Tancred and colleagues (Tancred *et al*., 2024) use a Health in All Policies approach to examine health worker responsiveness. They identify a lack of robust information systems and evidence to support the intersectoral decision-making processes for the health workforce and suggest that strengthening institutions of governance, and mechanisms of coordination, are both essential for improving the policymaking process, facilitating cross-sectoral spillovers and promoting effective leadership.

The articles selected for this special issue offer valuable perspectives on cross-sectoral analysis, highlighting both the progress made and gaps that remain in our understanding of intersectorality. Notably, contributions from the authors in this issue emphasize the importance of expanding system thinking beyond the traditional confines of the health care sector, advocating for a more integrated approach that better addresses the interconnected challenges faced by health systems. To this end, however, the issue clearly demonstrates the need for more robust case studies, reliable indicators and comparative analyses to fully grasp the complexities of intersectoral collaboration.
